# Trained immunity and related memory responses in lung health and disease

**DOI:** 10.3389/fimmu.2026.1937777

**Published:** 2026-09-15

**Authors:** Xin Sun, Dan Zhao, Lei Zhang, Jiao Liu, Ting Liu, Yao Liu, Anying Xiong, Jiahui Li, Qin Ran, Xiang He, Min Wu, Guoping Li, Junyi Wang

**Affiliations:** 1 College of Medicine, Southwest Jiaotong University, Chengdu, Sichuan, China; 2 Laboratory of Allergy and Precision Medicine, Department of Respiratory Medicine, Chengdu Institute of Respiratory Health, the Third People’s Hospital of Chengdu, Chengdu, Sichuan, China; 3 Clinical Medicine Department, North Sichuan Medical College, Nanchong, Sichuan, China; 4 State Key Laboratory of Quality Research in Chinese Medicine, Macau University of Science and Technology, Macao, Macao, SAR, China; 5 State Key Laboratory of Biotherapy, Sichuan University, Chengdu, Sichuan, China; 6 Wenzhou Institute, University of Chinese Academy of Sciences, Wenzhou, China

**Keywords:** alveolar macrophages, immunometabolism, lung diseases, pulmonary innate immunity, trained immunity

## Abstract

Immune memory has long been attributed to adaptive immunity, but trained immunity has broadened this view by showing that innate immune cells can retain altered function after prior stimulation and mount an enhanced response upon rechallenge. In the lung, prior local exposures can alter resident alveolar macrophages, whereas systemic immune stimulation can reprogramme bone-marrow progenitors and alter their myeloid output. Epithelial signals and alveolar-capillary barrier integrity help determine whether these changes protect tissue or promote injury. Reported metabolic pathways and chromatin features differ among cell types and experimental models; mTOR- and HIF-1α-mediated glycolysis and changes in H3K4me3 or H3K27ac can support altered innate responses. These responses may improve host defence or preserve tissue function without reducing pathogen burden, but can also contribute to barrier injury, chronic inflammation and fibrosis; in established tumours, ongoing local signals support immunosuppressive and angiogenic myeloid functions. We compare trained immunity in pulmonary myeloid cells, including trained alveolar macrophages, with other forms of alveolar macrophage memory, innate lymphoid memory-like responses and memory in pulmonary epithelial and stromal cells, and examine how these responses shape pulmonary infection, acute lung injury, chronic airway inflammation, pulmonary fibrosis and lung tumours.

## 1 Introduction

Pulmonary innate immunity operates at airway and alveolar surfaces that repeatedly encounter inhaled pathogens, allergens, pollutants, cigarette smoke and microbial components. An insufficient response permits pathogen expansion and dissemination, whereas an excessive response can damage the airway epithelium, alveolar epithelium, vascular endothelium, and alveolar-capillary barrier, ultimately impairing oxygenation (1–3). Airway epithelial cells form a physical barrier, sense inhaled stimuli, release cytokines and chemokines, and act together with alveolar macrophages (AMs), dendritic cells, and recruited myeloid cells to regulate local immune responses (2, 4).

Trained immunity refers to durable changes in innate immune function after prior stimulation. Innate immunity was once considered to lack long-term memory, but trained immunity shows that monocytes, macrophages, and their precursor cells can acquire durable functional changes after an initial stimulus and mount an enhanced functional response to homologous or heterologous stimuli after a resting interval (5–10). This response does not depend on antigen-specific receptors in T cells or B cells and is distinct from classical adaptive immune memory. These durable changes are supported by metabolic and epigenetic reprogramming and, in some settings, by changes in haematopoietic stem and progenitor cells (HSPCs) that alter myelopoiesis and the function of descendant myeloid cells (5, 7, 8, 11–19).

Peripheral blood monocyte studies alone cannot explain trained immunity in the lung. Peripheral blood monocytes and bone marrow HSPCs remain central to trained immunity research, but pulmonary responses also involve long-lived resident AMs and local airway exposure. Recent reviews have surveyed trained immunity across pulmonary infection, injury, repair and respiratory disease (20–22). Related syntheses have focused on macrophage memory in pulmonary disorders and post-infection lung tissue memory across immune and structural compartments (23, 24). Primary studies show that systemic immune stimulation and local airway exposure alter resident AM function and later pulmonary responses (25, 26).

Systemic HSPC training is well established, whereas the most direct tissue-resident evidence for pulmonary trained immunity comes from resident AM studies (15–19, 25–32). Natural killer (NK) cells and group 2 innate lymphoid cells (ILC2s) show memory-like responses, whereas airway epithelial cells and lung fibroblasts retain inflammatory or epigenetic memory; these responses remain distinct from myeloid trained immunity (33–41; **Figure 1**). We distinguish persistent functional changes in identified cell populations or lineages after stimulus withdrawal or recovery from responses observed only during ongoing exposure, and assess whether recall alters pathogen burden or a disease-specific lung outcome on rechallenge.

**Figure 1:**
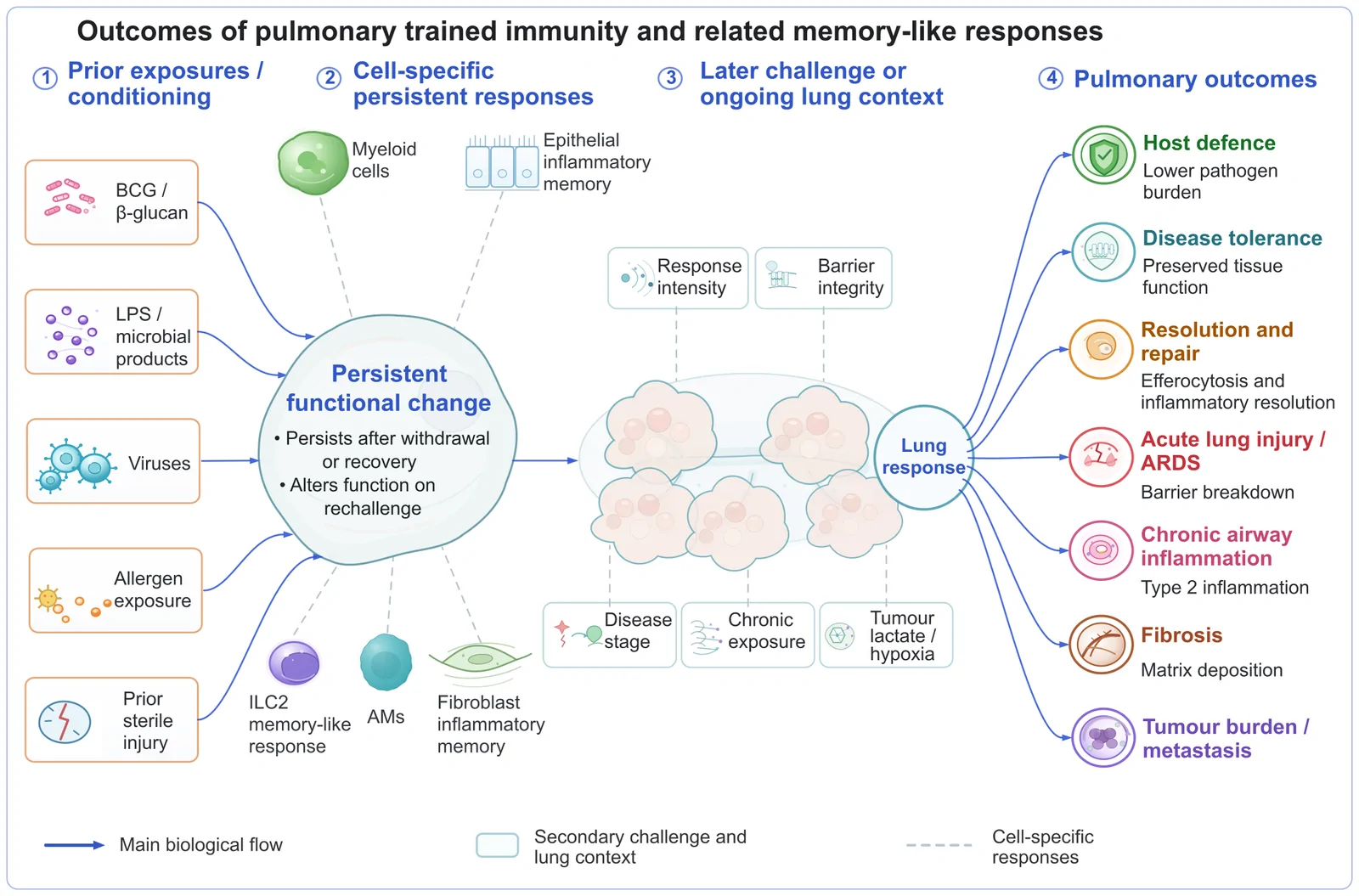
Outcomes of pulmonary trained immunity and related memory-like responses. Prior exposures or conditioning are linked to cell-specific persistent responses and pulmonary outcomes assessed after a later challenge; ongoing lung exposures are shown separately as context. BCG or β-glucan, LPS or other microbial products and respiratory viruses have been associated with persistent myeloid changes in model- and route-dependent settings (5, 25, 26, 31, 42–44). Chronic smoke exposure, ongoing fungal signalling and tumour-derived metabolic cues are treated as ongoing lung context rather than as prior conditioning (45–49). Allergen or IL-33 exposure supports ILC2 memory-like responses (37, 38, 50), and epithelial or fibroblast memory has been reported after infectious, allergic or inflammatory exposure (20, 33, 39–41). Pathogen burden and preserved tissue function are shown separately because improved pathogen control and disease tolerance are not interchangeable (51). The cell populations, lung contexts and pulmonary outcomes derive from distinct experimental systems; the branches do not imply that every cell population produces every outcome. Cellular sources are detailed in **Figure 2**, and molecular mechanisms in **Figure 3**. AM, alveolar macrophage; ARDS, acute respiratory distress syndrome; BCG, Bacillus Calmette–Guérin; IL, interleukin; ILC2, group 2 innate lymphoid cell; LPS, lipopolysaccharide.

## 2 Basic principles and lung-specific features of trained immunity

Sustained inflammatory activation alone does not define trained immunity. Conventional innate immune activation usually wanes once the stimulus is cleared. Trained immunity requires an initial stimulus followed by withdrawal or a resting interval and an enhanced functional response to homologous or heterologous rechallenge (5, 8–10, 52, 53). Relevant endpoints include cytokine production, phagocytic or killing capacity and antimicrobial activity. Increased TNF, IL-6 or activation-marker expression at a single time point is insufficient.

Metabolic or chromatin changes strengthen evidence for trained immunity when they persist after stimulus withdrawal and accompany an enhanced functional response upon rechallenge (5–8, 11–14). These measurements provide mechanistic support but do not replace evidence of altered function after rechallenge.

Trained immunity can also persist through reprogramming of HSPCs. By altering myeloid differentiation and the function of newly generated progeny, HSPC reprogramming can extend innate immune changes beyond the lifespan of peripheral monocytes (15–19).

Trained immunity and innate immune tolerance describe changes in cellular responsiveness after prior exposure. Trained immunity enhances selected functions on restimulation, whereas innate immune tolerance reduces selected responses (5–8, 14, 54, 55). Disease tolerance describes preservation of tissue function without necessarily lowering pathogen burden (56–58). β-glucan-induced neutrophil reprogramming during influenza illustrates that enhanced cellular recall can support disease tolerance (51). Enhanced or attenuated cellular recall does not by itself indicate whether pathogen control or tissue function improves.

Lung tissue state is central to the interpretation of pulmonary trained immunity. The alveolar space is chronically exposed to inhaled particles, low-dose microbial components, surfactant, and local metabolic signals, while the airway epithelium continuously senses pathogens, allergens, pollutants, and mechanical stimuli (1, 4, 27, 30, 34). Signals released by the airway epithelium directly influence myeloid cells and innate lymphoid cells (4).

Pulmonary studies differ in whether the initial stimulus is withdrawn before rechallenge and in which cells persistence or recall is measured. Evidence supports trained immunity when myeloid cells show an enhanced functional response to rechallenge after stimulus withdrawal or recovery; HSPC reprogramming linked to altered function in descendant myeloid cells supports central trained immunity (5, 8, 15–19, 52, 53). Recall responses in NK cells and ILC2s are generally described as memory-like; persistent epithelial or stromal responses are reported as inflammatory, epigenetic or fibrotic memory (33, 35, 37–41, 50, 59–62; **Table 1**). Studies of cigarette smoke, fungal Dectin-1 signalling and tumour-derived metabolic cues did not test persistence after stimulus withdrawal followed by functional rechallenge (45–49; **Table 2**).

**Table 1:** Operational criteria for distinguishing pulmonary trained immunity, innate immune tolerance, priming and related memory-like responses.

Response term or cell population	Operational definition	Key features supporting interpretation	Lung-relevant examples	Recommended wording
Trained immunity	Durable change in innate immune responsiveness after a primary stimulus, detected after a resting interval by an enhanced response to a secondary challenge	Prior stimulation; stimulus withdrawal or resting interval; enhanced response to secondary challenge; persistent functional change; preferably metabolic or epigenetic support	BCG-trained monocytes; β-glucan-trained myeloid cells; LPS-trained AMs	Use trained immunity when a primary stimulus is followed by stimulus withdrawal or a resting interval and a persistent functional change manifested as an enhanced response to a secondary challenge, preferably with metabolic or epigenetic evidence (5, 8, 11, 12, 26, 52, 53).
Central trained immunity	Trained immunity initiated in haematopoietic stem and progenitor cells, leading to biased myelopoiesis and altered function in descendant myeloid cells	HSPC transcriptional or chromatin remodelling; altered myeloid output; functional change in progeny	BCG-educated HSPCs; β-glucan-related myeloid and granulopoietic remodelling	Use central trained immunity when HSPC reprogramming is linked to altered myeloid output and function in descendant cells (15–19).
Memory responses in alveolar macrophages	Persistent changes in AM function after local or systemic exposure, described using the terminology of each study	Direct evidence that AMs carry the altered state; stimulus withdrawal or recovery; secondary challenge; functional readout; mechanistic support when available	LPS-trained AMs; BCG-associated memory AMs; post-viral antibacterial or antiviral AM memory	Use the source-specific term when a study demonstrates trained AMs, memory AMs, lung-resident memory macrophages or antiviral innate immune memory. Otherwise, describe the exposure, recovery interval and AM response after rechallenge directly; evidence from blood monocytes does not establish memory in AMs (25–31, 63).
Innate immune tolerance	Selective reduction in functional responsiveness after prior stimulation, demonstrated after an appropriate interval or recovery in a viable, defined cell population	Prior stimulation; appropriate interval or recovery; reduced response on rechallenge in a viable, defined cell population; exclusion of cell death, population shifts or general immunosuppression when relevant	LPS tolerance; sepsis-associated monocyte hyporesponsiveness after ex vivo restimulation	Use innate immune tolerance when prior stimulation produces selective low responsiveness on rechallenge after an appropriate interval or recovery. Distinguish it from disease tolerance, which preserves tissue function without necessarily improving pathogen control (14, 51, 55, 64–67).
Priming or acute activation	Short-lived inflammatory sensitization without evidence of durable memory after a resting interval	Immediate cytokine release; increase in activation markers; short-term sensitization without evidence of a resting interval or a persistent response upon rechallenge	Acute macrophage activation; transient epithelial alarm responses	Describe as priming or acute activation when evidence for a resting interval, rechallenge and persistent functional change is lacking (5, 8, 52, 53).
Memory-like response in NK cells	Enhanced NK-cell effector function after cytokine conditioning and a resting interval	Prior cytokine exposure; resting interval; enhanced IFN-γ production on restimulation	No lung-specific recall example is supported by the cited studies; the operational criterion is derived from cytokine-induced memory-like splenic NK cells (35).	Use a memory-like NK-cell response when prior cytokine conditioning is followed by a resting interval and enhanced NK-cell function on restimulation (35). Pulmonary NK-cell presence or antiviral function alone does not establish recall.
Memory-like response in ILC2s	Enhanced type 2 cytokine response after allergen or alarmin conditioning	Prior allergen, IL-33, or related alarmin exposure followed by persistence and a stronger IL-5 or IL-13 response; mechanistic support when available	Allergen-experienced ILC2s; neonatal IL-33-conditioned lung ILC2s; BRG1-dependent memory ILC2s	Use a memory-like ILC2 response as the general description when prior allergen or alarmin exposure is followed by a resting interval and stronger type 2 cytokine production on restimulation. Retain the terminology of individual studies, including ILC2 training, IL-33-experienced ILC2s or ILC2 memory (37, 38, 50, 59, 68).
Memory responses in pulmonary epithelial and stromal cells	Persistent inflammatory, epigenetic or repair-related state in structural cells after prior exposure	Stimulus withdrawal or resolution; persistent gene expression or chromatin state; altered function after rechallenge; cell-specific evidence when available	Epithelial memory of prior infection; papain-experienced adventitial stromal cells; RUNX1-associated epigenetic memory in lung fibroblasts	Use the term reported in the primary study when epithelial or stromal cells show persistent chromatin changes or an altered functional response after prior exposure. Otherwise, describe the exposure, recovery interval and response directly. Persistent chromatin marks without functional rechallenge do not establish trained immunity (33, 34, 39–41, 60–62).

Trained immunity, central trained immunity, innate immune tolerance and priming are response terms. The rows for AMs, NK cells, ILC2s, and pulmonary epithelial and stromal cells identify where persistence or recall was measured; the rows are not mutually exclusive. AM, alveolar macrophage; BCG, Bacillus Calmette–Guérin; BRG1, Brahma-related gene 1; HSPCs, haematopoietic stem and progenitor cells; IFN, interferon; IL, interleukin; ILC2, group 2 innate lymphoid cell; LPS, lipopolysaccharide; NK, natural killer; RUNX1, runt-related transcription factor 1.

**Table 2:** Outcomes after prior conditioning or during ongoing exposure across pulmonary disease settings.

Disease context	Prior conditioning or ongoing exposure and main cell populations	Protective effects	Pathological effects	Interpretive note	Key references
Respiratory infection	BCG, low-dose intranasal LPS, respiratory viral infection, mucosal bacterial vaccine, or β-glucan acting on AMs, monocytes, HSPC-derived myeloid cells, or neutrophils	Improved early microbial control and disease tolerance	Increased pneumococcal burden and lung injury after transfer of LPS-trained AMs in one mouse experiment	Evidence strength and outcome direction vary by model. Selected mouse studies demonstrate persistent functional changes in resident AMs; other studies assess monocytes, HSPC-derived myeloid cells or neutrophils.	(25, 26, 31, 32, 51, 56–58, 63, 72, 73)
Acute lung injury and ARDS	Prior conditioning/rechallenge: repeated LPS exposure; systemic β-glucan followed by LPS or the viral RNA analogue poly(I:C); *Pseudomonas aeruginosa* pneumonia followed by recovery and reinfection. During acute injury: viral RNA or DAMP-related signals acting on AMs, recruited monocytes and neutrophils.	Repeated LPS exposure: KLF4–MERTK-dependent AM efferocytosis; *Pseudomonas aeruginosa* re-exposure after 14 days of recovery: lower neutrophil accumulation and mortality	NLRP3 activation, IL-1β and IL-18 release, neutrophil recruitment, and alveolar-capillary barrier injury	These findings derive from experimental acute lung injury models and do not establish equivalent effects in clinical ARDS.	(3, 26, 42, 74)
Sepsis	Systemic infection, endotoxin exposure, or post-septic myeloid remodelling affecting circulating monocytes and bone marrow myeloid cells	—	Low mHLA-DR, impaired antigen presentation and weak cytokine responses in circulating monocytes; increased risk of secondary infection	Low mHLA-DR and reduced cytokine responses were measured in circulating monocytes, whereas enhanced TNF and IL-6 responses 12 weeks after caecal ligation and puncture were measured in bone-marrow monocytes	(55, 64–67, 75–77)
Chronic airway disease	During smoke exposure: cigarette smoke acting on AMs, recruited macrophages and epithelial cells. Prior conditioning or inflammatory imprinting: allergen or alarmin exposure involving macrophages derived from bone marrow or blood monocytes, ILC2s and epithelial or stromal cells.	—	Impaired AM phagocytosis, monocyte-derived macrophage recruitment, NLRP3–GSDMD activation, TNF-dependent macrophage inflammatory memory, type 2 inflammation, mucus production, and airway remodelling	Smoke studies assessed ongoing exposure. The asthma macrophage study identified persistent responses in progenitor-derived macrophages but did not establish resident AM memory; ILC2 and stromal studies tested recall after conditioning and restimulation.	(33, 34, 37, 38, 46, 48–50, 59, 61, 68, 78)
Pulmonary fibrosis	Prior conditioning/rechallenge: systemic or intranasal β-glucan exposure before bleomycin injury; resolved bleomycin injury followed by reinjury in experiments testing fibroblast memory. During fibrosis: Dectin-1-related fungal signalling.	Systemic β-glucan reduced collagen deposition; intranasal β-glucan improved survival, lowered disease scores and reduced weight loss without reducing fibrotic area	Ongoing fungal Dectin-1–Raf1 signalling, RUNX1-associated fibroblast reactivation after reinjury and ECM deposition	The β-glucan studies separated initial exposure from later bleomycin injury; fibroblast memory was tested after resolution and reinjury; the Dectin-1 study examined ongoing fungal signalling.	(43, 47, 62, 79)
Lung tumour immunity and metastasis	Prior conditioning: β-glucan exposure involving lung interstitial macrophages or trained granulopoiesis with antitumour neutrophil activity; influenza exposure involving resident AMs. In established tumours: lactate or hypoxia acting on TAMs	Increased macrophage TNF production, phagocytosis and cytotoxicity, with reduced metastasis	Lactate-induced HIF-1α-dependent VEGF and ARG1 expression and other immunosuppressive or angiogenic TAM functions	Studies of tumour metabolites did not test previously trained cells; direct clinical extrapolation remains unsupported	(44, 45, 71, 77, 80–84)

AM, alveolar macrophage; ARDS, acute respiratory distress syndrome; ARG1, arginase 1; BCG, Bacillus Calmette–Guérin; DAMP, damage-associated molecular pattern; ECM, extracellular matrix; GSDMD, gasdermin D; HIF-1α, hypoxia-inducible factor 1α; HSPCs, haematopoietic stem and progenitor cells; IL, interleukin; ILC2, group 2 innate lymphoid cell; KLF4, Krüppel-like factor 4; LPS, lipopolysaccharide; MERTK, MER proto-oncogene tyrosine kinase; mHLA-DR, human leukocyte antigen-DR expression on monocytes; NLRP3, NOD-, LRR- and pyrin domain-containing protein 3; poly(I:C), polyinosinic:polycytidylic acid; RUNX1, runt-related transcription factor 1; TAMs, tumour-associated macrophages; TNF, tumour necrosis factor; VEGF, vascular endothelial growth factor.

## 3 Cellular sources and related memory-like states

### 3.1 Alveolar macrophages

AMs provide the clearest tissue-resident myeloid evidence for pulmonary trained immunity. AMs reside long term in the alveolar space, where they are continuously exposed to inhaled particles, microbial components, surfactant, and dying cells. They support pathogen clearance, efferocytosis, control of inflammation and surfactant homeostasis (2). This anatomical position distinguishes AMs from peripheral monocytes recruited into the lung. Prior stimulation may alter AM phagocytosis, antiviral priming, inflammatory threshold or suppressive function without requiring a uniform increase in cytokine release (20, 25–27, 31, 42).

Parenteral BCG can alter resident AM function. In mice, a trained AM phenotype was not detected 2 weeks after subcutaneous BCG vaccination. By 5 weeks, lung-resident AMs showed higher major histocompatibility complex class II (MHC II) expression and enhanced cytokine release after restimulation; separate assays at 8 weeks demonstrated increased glycolysis. AMs from vaccinated mice controlled *Mycobacterium tuberculosis* more efficiently ex vivo. Lineage and depletion experiments indicated that induction of memory AMs was not explained by replacement with circulating monocytes, and gut–lung signals were implicated in its induction (25). Respiratory viral models demonstrate related but distinct AM states. After adenoviral infection, memory AMs showed higher MHC II expression, increased glycolytic metabolism and greater production of neutrophil-recruiting chemokines upon restimulation; their formation and maintenance were independent of monocytes or bone-marrow progenitors (63). Following recovery from SARS-CoV-2 infection, resident AMs exhibited epigenetic imprinting (31).

Local exposure can train resident AMs directly. In a low-dose intranasal LPS model, neutrophilic inflammation had resolved before AMs were isolated on day 6. These AMs produced more cytokines and showed greater phagocytosis after ex vivo *Streptococcus pneumoniae* challenge, and enhanced IL-6 recall remained detectable at 2 and 6 weeks. PKH26 labelling did not show replacement of the resident AM pool by recruited monocytes at either 24 h or 6 days after LPS exposure (26).

The low-dose intranasal LPS, parenteral BCG and respiratory viral studies used different conditioning routes, intervals and secondary challenges, and the original reports described trained AMs, memory AMs, lung-resident memory macrophages or antiviral innate immune memory (25, 26, 31, 63). Equivalence among the reported AM phenotypes has not been established. Prolonged STAT1 binding after IFN-γ priming may support macrophage recall, but that study did not test pulmonary AM training (69).

### 3.2 Dendritic cells

In cryptococcal vaccination models, early IFN-γ signalling and recruited monocyte-derived dendritic cells (DCs) support STAT1-dependent Th1 responses, T-cell recruitment and fungal clearance (70). These experiments establish a role for DCs in vaccine-induced protection but do not demonstrate durable training in DCs.

### 3.3 Monocytes and neutrophils

Pulmonary trained immunity also reflects myeloid programming initiated in the bone marrow. BCG and β-glucan can act on HSPCs, altering myeloid differentiation bias and the function of progeny cells (15, 16, 19). In a human study, BCG vaccination induced sustained transcriptional programmes in bone marrow HSPCs related to myeloid development and function (17). Single-cell studies further show that BCG vaccination can alter gene expression and chromatin accessibility in human bone marrow progenitors and influence peripheral innate immune responses (18). Together, these human studies support central trained immunity by linking HSPC transcriptional and chromatin remodelling to altered peripheral innate responses.

During lung infection or injury, large numbers of monocytes and neutrophils enter lung tissue from the circulation. If these cells originate from trained HSPCs, they may already have different inflammatory responses or antimicrobial functions before they reach the lung (15–19). BCG-induced HSPC remodelling can enhance the antimicrobial function of progeny myeloid cells (16). β-glucan training can also alter myelopoiesis and neutrophil function (15, 71).

HSPC-derived monocytes and neutrophils enter a lung environment shaped by epithelial signals, surfactant, dying cells and local metabolites. Pulmonary responses may therefore reflect both recruited cells and resident AMs, but most studies have not quantified their respective contributions in the same model (15–19, 25, 26, 31).

### 3.4 NK cells and ILC2s

During respiratory infection, NK cells can enter lung tissue, recognise virus-infected cells and produce IFN-γ, chemokines and cytotoxic molecules (36). These acute responses contribute to early antiviral defence but may also worsen tissue injury when inflammation is excessive; they do not by themselves demonstrate persistent recall. Direct evidence for cytokine-induced NK-cell memory-like recall derives mainly from a non-pulmonary model in which splenic NK cells were conditioned briefly with IL-12 and IL-18 in the presence of low-dose IL-15 as a survival factor; after adoptive transfer, they produced more IFN-γ upon restimulation 1–3 weeks later (35).

Lung ILC2s respond to epithelial-derived alarmins such as IL-33 and IL-25 by producing IL-5 and IL-13, which promote eosinophil recruitment, mucus secretion and type 2 inflammation. After allergen exposure, ILC2s produced stronger type 2 cytokine responses to repeated or heterologous allergen stimulation (37). In a papain model, memory ILC2s remained detectable 2 months after priming and responded to low-dose IL-33 (59). These studies support persistent ILC2 recall after conditioning and restimulation. Their conditioning and recall protocols differ, and the evidence does not establish that all of these ILC2 responses represent trained immunity (37, 38, 50, 59).

### 3.5 Pulmonary epithelial and stromal cells

Airway epithelial cells can retain inflammatory memory after allergic inflammation (33) and inflammatory or epigenetic changes after prior bacterial or viral exposure (39, 40, 60). After pre-exposure to *Pseudomonas aeruginosa* flagellin, human bronchial epithelial cells showed altered inflammatory responses to later unrelated stimuli; in the *Aspergillus fumigatus* rechallenge assay, inhibition of histone acetyltransferase activity or H3K9 methylation reduced the enhanced IL-8 response (39). Repeated rhinovirus infection can also induce changes in DNA methylation and gene expression in human bronchial epithelial cells, suggesting that viral exposure can leave relatively persistent epigenetic marks in the epithelial layer (40). In the A549 human lung adenocarcinoma cell line, *Streptococcus pneumoniae*-induced H3K4me2 persisted for 7–9 days after antibiotic clearance and through two passages; secondary infection altered transcription, metabolism and lysosomal transport and increased bacterial adhesion and cellular permissiveness. Stimulus withdrawal and functional rechallenge were demonstrated in cultured cells; the mouse study detected infection-associated epithelial H3K4me2 without a cell-specific *in vivo* recall assay (60).

Lung fibroblasts and stromal cells can also develop inflammatory memory responses. Human lung fibroblasts pre-exposed to poly(I:C), rested for 24 h and then restimulated with TNF showed increased *IL6* expression and IL-6 release, indicating a retained functional change after inflammatory stimulation (41). In mice, papain increased accessibility of the *Ccl11* locus in adventitial stromal cells through IL-4 and IL-13; weeks later, these cells showed an enhanced type 2 recall response. Induction of an opposing type 1 response during the first challenge blocked both *Ccl11* accessibility and later eosinophilic recall through IFN-γ-dependent cross-regulation (61). In a bleomycin reinjury model, lineage-labelled lung mesenchymal cells largely reversed their initial transcriptional programme after resolution, but a subset of regions formerly bound by runt-related transcription factor 1 (RUNX1) remained accessible and the second injury produced an enhanced fibrotic response. RUNX1 knockdown reduced fibrotic mesenchymal cell activation immediately after cell isolation (62). These studies examine non-haematopoietic epithelial or stromal cells and differ in whether they demonstrate persistent chromatin or transcriptional changes, altered function after restimulation or both (33, 39–41, 60–62). **Figure 2** summarizes bone marrow programming, circulating myeloid progeny and local lung signals.

**Figure 2:**
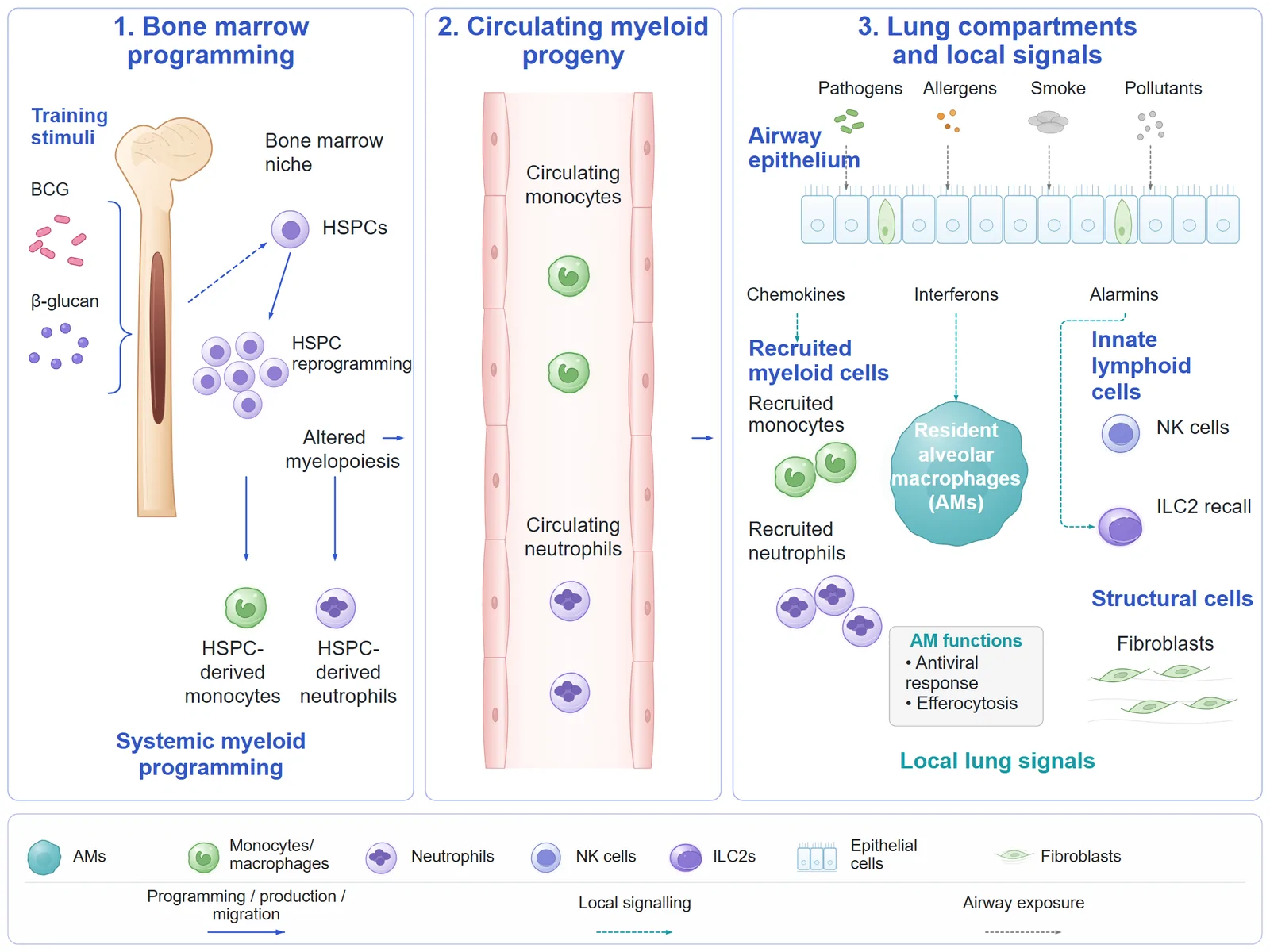
Bone marrow programming, circulating myeloid progeny and local lung signals. Systemic BCG or β-glucan exposure can reprogramme haematopoietic stem and progenitor cells (HSPCs) and alter myeloid output (15–18). Resident alveolar macrophages (AMs) are shown separately from recruited monocytes and neutrophils. Interferon effects on AMs are model-specific (26, 31, 63). Epithelial alarmins are associated with group 2 innate lymphoid cell (ILC2) recall (37, 38, 50, 59), whereas epithelial chemokines support myeloid recruitment (4). NK cells, ILC2s and structural cells are shown as parallel populations rather than pathways downstream of AM training. AM, alveolar macrophage; BCG, Bacillus Calmette–Guérin; HSPC, haematopoietic stem and progenitor cell; ILC2, group 2 innate lymphoid cell; NK, natural killer.

## 4 Disease setting determines the outcomes of trained immunity and related memory-like states

### 4.1 Respiratory infection

Evidence for a protective role of pulmonary trained immunity is strongest in respiratory infection. In humans, BCG vaccination induced epigenetic and functional reprogramming in circulating monocytes and reduced viraemia during experimental viral infection; these findings support systemic trained immunity but do not directly demonstrate a lung-resident AM state (9, 32, 72). In mouse lung models, subcutaneous BCG generated resident AMs with altered recall function and improved early pulmonary control of *Mycobacterium tuberculosis*, and adenovirus-induced memory AMs improved later pneumococcal control (25, 63). By contrast, post-SARS-CoV-2 AM memory ameliorated subsequent influenza disease without lowering viral titre, and β-glucan-induced neutrophil reprogramming limited tissue injury and preserved lung function during influenza without reducing viral burden; both findings support disease tolerance rather than improved pathogen clearance (31, 51).

Mucosal vaccination can also induce heterologous protection. In mice, the inactivated polyvalent vaccine MV130 alters the pulmonary immune environment and provides long-term protection against experimental respiratory viral infection. MV130 also protected *Rag1*-deficient mice against systemic candidiasis, supporting an innate contribution to heterologous protection (73).

### 4.2 Acute lung injury and ARDS

In experimental acute lung injury, prior conditioning can worsen or limit tissue damage depending on the stimulus and myeloid population involved (26, 42, 74). In the low-dose intranasal LPS model, adoptive transfer of trained AMs increased pneumococcal burden and lung injury, whereas intact LPS-pre-exposed mice cleared bacteria more efficiently (26). Systemic β-glucan reprogrammed AMs and worsened LPS- or poly(I:C)-induced lung injury (42).

By contrast, in another LPS protocol, pre-exposure followed by a second dose 7 days later expanded MERTK^hi^ resident AMs, whose KLF4–MERTK-dependent efferocytosis enhanced resolution of lung injury. In a distinct *Pseudomonas aeruginosa* protocol, lethal rechallenge after sublethal pneumonia and 14 days of recovery produced less neutrophil accumulation and lower mortality; transfer of previously exposed AMs 4 h after lethal infection reduced histological injury and lung neutrophils at 24 h (74).

### 4.3 Sepsis

Post-sepsis myeloid dysfunction can increase susceptibility to secondary pulmonary infection. Clinical studies describe reduced antigen presentation and ex vivo cytokine responses in circulating monocytes, whereas experimental work has identified persistent hyperresponsiveness in bone-marrow monocytes (64–67, 75).

Endotoxin tolerance provides an important mechanism for understanding immune paralysis. Classical studies of LPS tolerance show that macrophages do not suppress all genes equally upon re-exposure. Gene-specific chromatin regulation leaves some inflammatory genes less responsive while antimicrobial or regulatory genes remain inducible (55). This selective reprogramming supports tolerance as an innate memory state, which in sepsis can appear as low monocyte or macrophage responsiveness and contribute to immune paralysis (55, 64–67).

Clinically, reduced human leukocyte antigen-DR expression on monocytes (mHLA-DR) is commonly used as a marker of sepsis-associated immune paralysis. Low mHLA-DR is associated with reduced monocyte cytokine responses to ex vivo endotoxin stimulation and increased risks of secondary infection and death (64). Because mHLA-DR is measured in circulating monocytes, it does not establish the functional state of resident AMs.

In mice examined 12 weeks after caecal ligation and puncture, bone-marrow monocytes produced more TNF and IL-6 after 24 h ex vivo LPS stimulation and showed increased glycolytic activity and persistent transcriptional changes; the original study described this phenotype as a long-lasting state of trained immunity (75). Studies targeting IL-4 to myeloid cells further show that modulation of trained immunity can reverse sepsis-associated immune paralysis and partly restore immune function in experimental models and human samples (76). These studies assessed different cell populations at different times after sepsis and therefore do not establish that low and enhanced responsiveness coexist in the same host or cell population.

### 4.4 Chronic airway inflammatory diseases

Chronic obstructive pulmonary disease (COPD) and asthma involve different innate immune changes rather than a single trained immunity mechanism. Cigarette smoke, pollutants and repeated infection drive myeloid remodelling in COPD, whereas asthma includes TNF-dependent macrophage inflammatory memory, epithelial alarmin–ILC2 memory-like responses and epithelial or stromal inflammatory memory (33, 34, 37, 38, 46, 48–50, 78).

In COPD, cigarette smoke can increase macrophage glycolysis and reactive oxygen species, impair mitochondrial respiration and reduce bacterial phagocytosis by AMs (46). Single-cell studies further show neutrophil infiltration and recruitment of monocyte-derived AMs with pro-inflammatory gene signatures associated with emphysema-like tissue remodelling (48).

NLRP3 inflammasome signalling contributes to smoke-induced lung inflammation. Cigarette smoke activates NLRP3-dependent GSDMD in bronchial epithelial cells and bronchoalveolar macrophages, promoting IL-1β release and inflammatory cell recruitment. Deficiency of NLRP3, caspase-1/11 or GSDMD reduces cigarette-smoke-induced lung inflammation (49).

Long-term innate immune changes in asthma involve both myeloid and non-myeloid compartments. In house dust mite (HDM)-induced allergic airway inflammation, macrophages differentiated from bone marrow of HDM-allergic mice or blood monocytes of patients with asthma showed inflammatory transcriptional reprogramming and increased mediator production upon stimulation. Mouse and *in vitro* experiments additionally identified metabolic activation, and TNF blockade or deletion of *Tnf* in myeloid cells prevented inflammatory imprinting in bone-marrow-derived macrophages from HDM-allergic mice. These findings support TNF-dependent macrophage inflammatory memory in asthma but do not establish autonomous memory in resident pulmonary AMs (78). Prior allergen or alarmin exposure can intensify eosinophil recruitment, mucus secretion and airway hyperresponsiveness through ILC2 memory-like responses, while cytokine cross-regulation during the initial response can enhance or suppress later stromal eosinophilic recall (37, 38, 50, 59, 61).

The cigarette-smoke studies measured macrophage dysfunction or inflammatory signalling during ongoing exposure and did not test persistence after smoke withdrawal followed by rechallenge (46, 48, 49). By contrast, the ILC2 and stromal-cell studies separated initial allergen or cytokine exposure from a later challenge and measured altered recall responses (37, 38, 61).

### 4.5 Pulmonary fibrosis

After lung injury, AMs, monocyte-derived macrophages, epithelial cells, fibroblasts and neutrophils contribute to the clearance of dying cells, barrier repair and matrix remodelling (20, 43, 47). Because collagen deposition is a tissue-level outcome of these multicellular processes, a change after prior exposure does not identify the conditioned cell population or establish a persistent functional response to a later challenge (52, 53).

Systemic β-glucan pretreatment reduced mortality and collagen deposition after bleomycin injury in mice. Prior β-glucan exposure increased lung neutrophil accumulation, AM efferocytosis and histone changes in AMs and neighbouring epithelium. Efferocytosing macrophages released resolvin D1 (RvD1) and other soluble mediators that induced epithelial sirtuin 1 (SIRT1) and reduced apoptosis, linking a conditioned myeloid response to inflammatory resolution and less fibrosis (43).

A single low-dose intranasal β-glucan exposure generated CCR2-dependent monocyte-derived ApoE^+^CD11b^+^ AMs that peaked on day 7 and remained detectable on day 21. Cells collected from bronchoalveolar lavage fluid on day 5 and transferred to untreated recipients retained increased IL-6 production after ex vivo LPS restimulation 48 h later, supporting a cell-intrinsic functional change; persistence of this function to day 21 was not tested. Bleomycin administered 7 days after β-glucan improved survival, lowered disease scores and reduced weight loss over 14 days without reducing fibrotic lung area (79).

Ongoing fungal signalling promoted fibrosis. Dectin-1 expression on AMs correlated with fibrotic severity in human and mouse lung tissue; inhibiting Dectin-1 or Raf1 reduced fibrosis in mice and lowered profibrotic mediator production by human AMs and fibroblasts (47).

The β-glucan and Dectin-1 studies test different processes. Both β-glucan studies separated pre-exposure from bleomycin challenge, whereas the Dectin-1 study examined ongoing fungal signalling during fibrosis (43, 47, 79). In the bleomycin reinjury model, persistent RUNX1-associated chromatin accessibility in lung mesenchymal cells accompanied an enhanced fibrotic response, supporting epigenetic memory in these cells (62).

### 4.6 Lung tumour immunity and metastasis

Prior β-glucan or influenza exposure can generate antitumour myeloid responses in experimental pulmonary tumour models, whereas tumour-derived metabolites and cytokines act on tumour-associated macrophages (TAMs) within established tumours (44, 45, 71, 80, 81). Current evidence comes mainly from experimental metastasis and post-viral AM models (44, 71, 80, 82, 83).

β-glucan training provides relatively direct evidence that myeloid cells can participate in antitumour responses. Training with whole β-glucan particles (WGP) made lung interstitial macrophages more responsive to tumour-derived signals, with increased TNF production, phagocytosis and cytotoxicity against tumour cells. These changes reduced lung metastasis and prolonged survival (80). β-glucan-induced training of granulopoiesis can also enhance the antitumour activity of neutrophils and restrict tumour progression (71). The lung interstitial macrophage experiments used experimental metastasis models in which WGP treatment preceded tumour-cell inoculation (80).

Respiratory influenza can also train lung-resident AMs for antitumour activity. In mice, influenza A virus generated long-term lung-specific antitumour immunity that depended on resident AMs rather than systemic inflammation or replacement by bone-marrow-derived cells (44). A subsequent study showed that influenza A virus infection left transcriptional imprints of acute type I interferon signalling in resident AMs. Type I interferon signalling was required for training-induced antitumour activity but limited training-induced antibacterial activity in both infected mice and an ex vivo cytokine-mediated training system (84).

Tumour-derived lactate promotes VEGF and ARG1 expression in TAMs through HIF-1α-dependent signalling (45). TAMs can also inhibit antitumour immunity through immunosuppressive and angiogenic mediators and T-cell suppression (81). Studies of lactate and hypoxia examined TAMs within established tumours; previously trained AMs or monocytes were not tested.

## 5 Mechanisms shaping pulmonary trained immunity and related memory-like states

In human monocytes, β-glucan induces mTOR- and HIF-1α-mediated glycolysis, glutaminolysis and fumarate accumulation; fumarate inhibits KDM5 demethylases and increases H3K4me3 at pro-inflammatory cytokine promoters (11, 12). Other reported features include H3K27ac, H3K4me1, chromatin accessibility and DNA methylation, but no single mark defines trained immunity (5, 13, 18). Chromatin remodelling in HSPCs can link persistent progenitor changes to biased myelopoiesis and altered function in descendant myeloid cells (15–19).

Low-dose LPS training of AMs requires type I interferon signalling, fatty acid oxidation and glutaminolysis despite limited baseline chromatin remodelling (26). Post-SARS-CoV-2 AM memory also involves type I interferon signalling but includes epigenetic imprinting (31). IFN-γ is required to induce, but not maintain, AM memory after adenovirus infection (63). BRG1 increased *Hif1a* enhancer accessibility in memory ILC2s and supported glycolytic metabolism, whereas ILC2-specific *Smarca4* deletion reduced the secondary response to low-dose IL-33 (59). Epithelial H3K4me2 accompanied altered responses to reinfection, IL-4/IL-13-dependent *Ccl11* accessibility supported stromal type 2 recall, and RUNX1-associated accessible chromatin accompanied fibroblast reactivation after reinjury (60–62).

IL-33 supports ILC2 recall (38, 50), and RvD1–SIRT1 signalling accompanies epithelial protection after β-glucan conditioning (43). Ongoing Dectin-1–Raf1 signalling and tumour lactate or hypoxia act during ongoing disease, without evidence of recall after stimulus withdrawal (45, 47, 81). **Figure 3** maps these mechanisms and signals to the cells and models in which they were reported.

**Figure 3:**
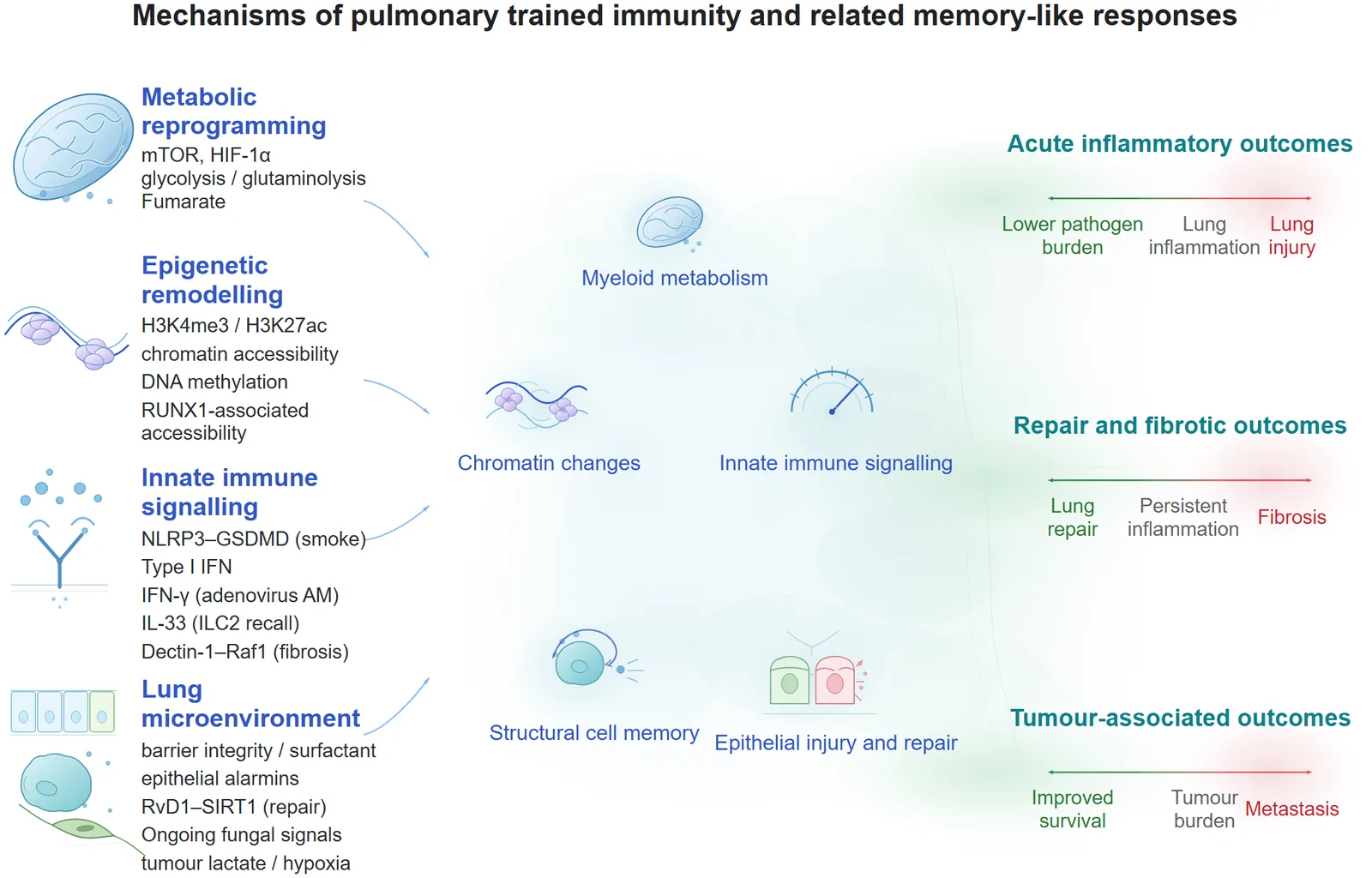
Mechanisms of pulmonary trained immunity and related memory-like responses. Metabolic and chromatin features are shown as reported in distinct cells and models rather than as a common mechanism across lung compartments (11–13, 18, 26, 31, 33, 40, 59–62). Type I interferon (IFN) is shown in alveolar macrophages (AMs) trained with low-dose LPS and in post-SARS-CoV-2 AM memory, IFN-γ in adenovirus-experienced AMs, and interleukin-33 (IL-33) in group 2 innate lymphoid cell (ILC2) recall (26, 31, 38, 50, 63); NLRP3–GSDMD is shown during ongoing cigarette smoke exposure (49). RvD1–SIRT1, ongoing Dectin-1–Raf1 signalling and RUNX1-associated accessibility are shown only in their respective fibrosis or reinjury models (43, 47, 62). Barrier integrity, surfactant, epithelial alarmins, ongoing fungal signals and tumour lactate or hypoxia are shown as local conditions rather than as a shared induction mechanism (1–4, 20, 45, 47, 81). The bidirectional gradients indicate context-dependent shifts between protective or reparative and pathological outcomes across distinct experimental models; they do not represent a temporal sequence within a single model. Detailed pulmonary outcomes are compared in **Figure 1** and **Table 2**. AM, alveolar macrophage; GSDMD, gasdermin D; H3K4me3, trimethylation of histone H3 lysine 4; H3K27ac, acetylation of histone H3 lysine 27; HIF-1α, hypoxia-inducible factor 1α; IFN, interferon; IL, interleukin; ILC2, group 2 innate lymphoid cell; LPS, lipopolysaccharide; mTOR, mechanistic target of rapamycin; NLRP3, NOD-, LRR- and pyrin domain-containing protein 3; RvD1, resolvin D1; RUNX1, runt-related transcription factor 1; SIRT1, sirtuin 1.

## 6 Unresolved questions and perspective

Persistence and reversibility differ across pulmonary cell types and cannot be inferred from a molecular mark alone. Experimental AM states can persist for weeks, memory ILC2s for months, epithelial H3K4me2 through bacterial clearance and cell passages, and stromal or fibroblast states beyond resolution of the initial inflammation or injury (25, 26, 31, 59–63). These intervals are not directly comparable, and most evidence remains preclinical. Studies should test whether these states can be erased without impairing baseline host defence (5, 8, 52, 53).

Future studies should distinguish resident from recruited cell populations at each disease stage. Lineage tracing, selective depletion and bone-marrow transplantation should be combined with secondary-challenge experiments to determine whether altered function resides in AMs, newly recruited myeloid cells or structural cells within the same experimental system.

Lower pathogen burden, improved survival and reduced histological injury are not interchangeable endpoints. Infection studies should measure pathogen burden together with tissue-injury and recovery endpoints on the same time axis (26, 31, 51). Bleomycin, allergen and tumour studies should instead report fibrosis, airway physiology or tumour burden together with the functional response of the conditioned cell population.

To distinguish systemic HSPC reprogramming from local lung remodelling, bone marrow, peripheral blood and lung tissue should be sampled on the same time axis. Relevant local exposures, including epithelial signals, surfactant, smoke, allergens or tumour metabolites, should be measured or experimentally controlled.

Single-cell and spatial profiling can identify candidate cell populations and their anatomical location; when sampled longitudinally, these methods can also document population-level molecular persistence. By themselves, however, they cannot establish cell-intrinsic persistence or altered function upon rechallenge. Such datasets should be combined with metabolic or epigenetic measurements and functional rechallenge in the same defined population (5, 8, 10, 52, 53). Human lung cell atlases and spatial transcriptomic resources can guide the selection and localisation of candidate populations (85–87).

Clinical translation also requires cell-, route- and disease-specific safety testing. Systemic β-glucan worsened LPS- or poly(I:C)-induced lung injury in mice, whereas other β-glucan models improved influenza disease tolerance or bleomycin outcomes (42, 43, 51), so protective effects cannot be generalised across stimuli or disease settings. The risks of local versus systemic delivery, particularly in chronic inflammatory or autoimmune lung disease, remain untested; direct clinical evidence that trained immunity inducers worsen autoimmunity is insufficient. Route, dose, duration, target cell, reversibility and inflammatory endpoints should be defined before efficacy testing; pulmonary administration of BCG or β-glucan remains investigational (21, 42, 77, 88).

## 7 Conclusions

Evidence for pulmonary trained immunity is most direct in resident AM studies that separate conditioning from recovery and test AM function after rechallenge; HSPC reprogramming is well supported, but direct tracing of reprogrammed progeny to lung-specific outcomes remains limited. Evidence for NK-cell recall derives from cytokine-conditioned splenic cells, whereas lung models support ILC2 recall and epithelial or stromal memory; these responses in lymphoid and non-haematopoietic cells remain distinct from myeloid trained immunity. Cell-specific recall can accompany protection or injury, depending on its effects on pathogen burden and the relevant tissue or disease endpoint.

Direct human lung evidence remains limited, particularly for linking changes that persist after recovery to altered function upon rechallenge in a defined pulmonary cell population or cellular lineage.
